# G-protein-coupled receptor GPR17 inhibits glioma development by increasing polycomb repressive complex 1-mediated ROS production

**DOI:** 10.1038/s41419-021-03897-0

**Published:** 2021-06-12

**Authors:** Huiqing Liu, Rui Xing, Zhimin Ou, Junying Zhao, Guolin Hong, Tong-Jin Zhao, Ying Han, Ying Chen

**Affiliations:** 1 State Key Laboratory of Cellular Stress Biology, School of Life Sciences, Xiamen University, Xiamen, Fujian Province China; 2 The Department of Laboratory Medicine, The First Affiliated Hospital of Xiamen University, Xiamen, Fujian Province China; 3 Xiamen Key Laboratory of Genetic Testing, Xiamen, Fujian Province China; 4 Institute of Metabolism and Integrative Biology, Fudan University, Shanghai, China

**Keywords:** CNS cancer, CNS cancer

## Abstract

Glioma is the most common primary tumor in the central nervous system. However, the development of glioma and effective therapeutic strategies remain elusive. Here, we identify GPR17 as a potential target to treat glioma. Data mining with human LGG and GBM samples reveals that GPR17 is negatively correlated with glioma development. Overexpressing GPR17 inhibits glioma cell proliferation and induces apoptosis by raising ROS levels. GPR17-overexpressing glioma cells are less tumorigenic in the brain than in control cells. Mechanistically, GPR17 inhibits the transcription of *RNF2*, a key component in the PRC1 complex, through cAMP/PKA/NF-κB signaling, leading to reduced histone H2A monoubiquitination. ChIP-Seq and RNA-Seq analyses reveal *KLF9* as a direct target of RNF2. KLF9 mediates the functions of GPR17 and RNF2 in glioma cells. Furthermore, activation of GPR17 by its agonist inhibits glioma formation. Our findings have thus identified GPR17 as a key regulator of glioma development and a potential therapeutic target for gliomas.

## Introduction

Glioma is one of the most prevalent primary tumors in central nervous system. Histologically, gliomas have similar characteristics as glial cells. According to World Health Organization (WHO) classification, gliomas were categorized into groups, including astrocytic, neuronal, and mixed neuronal–glial tumors [1]. During development, gliomas were divided into four grades, according to their malignancies [2]. Grade IV glioma (glioblastoma multiforme, GBM) is the most common and lethal tumor in the brain. GBM patients usually survive no longer than 15 months after diagnosis [3–5]. At present, chemotherapy, radiotherapy, and surgery represent the primary therapeutic interventions for GBM, but none of these interventions showed satisfying prognosis [3]. In the past years, researchers were dedicated to study the biology of gliomas, and have discovered many genes or pathways that were crucial in glioma development [6–9]. However, it is still far from fully understanding the molecular basis underpinning the glioma development, and effective therapeutic interventions, especially pharmacological treatment remain greatly in need.

G-protein coupled receptors located on the cell surface and formed the largest membrane protein family. Their regulations on numerous physiological processes and high accessibilities for ligands made them one of the most popular protein families as drug targets [10, 11]. Through data mining on the online database (https://www.cancer.gov/tcga), we found that GPCR family may play a role in the giloma development with notable significance. Although the effects of GPCRs in glioma have emerged [12, 13], whether GPCRs could serve as drug targets in glioma remained elusive.

G protein-coupled receptor 17 (GPR17) belongs to G-protein-coupled receptor family. It responded to both uracil nucleotides and cysteinyl leukotrienes (cysLTs) [14]. Previously, GPR17 has been established to play important roles in oligodendrocyte development and metabolism. During oligodendrocyte and myelin sheath development, GPR17 acted as a negative regulator [15, 16]. It inhibited myelinogenesis through both inhibiting oligodendrocyte differentiation and promoting apoptotic cell death [16]. Moreover, GPR17 was found to control glycolysis and lactate production in oligodendrocytes [17]. Although *GRP17* was found to be expressed in glioma [18–20], whether and how GPR17 was involved in the glioma development remain largely unknown.

In our study, using multiple approaches, we revealed the inhibitory role of GPR17 in glioma development, and demonstrated the pathway and the key molecules that mediated its biological effects in glioma cells. Using GPR17 agonist to activate GPR17 activity, we discovered that GPR17 had a therapeutic potential in glioma treatment.

## Materials and methods

### Stable cell lines generation

The glioma cell lines U87MG and U251 were obtained from the Institute of Cell Biology, Chinese Academy of Sciences (Shanghai) and cultured in Dulbecco’s modified Eagle’s medium (BasalMedia, Cat# L110KJ) supplemented with 10% fetal bovine serum (FBS). GPR17-overexpressing stable cell lines were generated in U87MG and U251 cells. Cells were transfected with control or GPR17-overexpressing constructs (pLV03 vector), using liposomes (Yeasen, Cat# 40802ES02) following the manufacturer’s instruction, and screened by blastidicin (4 μg/ml) until monoclonal cell line was obtained. To generate GPR17-knockdown cell lines, U87MG and U251 cells were transfected with scramble or GPR17 shRNA constructs (pLKO1 vector), and then screened by puromycin (1 μg/ml). RNF2-overexpressing/knockdown cell lines were generated with the same protocol. Both pLV03 and pLKO1 vectors were kindly provided by Dr. Zhao Tong-Jin’s lab.

For the intracranial model, control or U87-GPR17 cells were transfected with pLenti-CBh-3FLAG-luc2-tCMV-mNeonGreen-F2A-Puro (OBiO, Cat# H7656), and then were screened by 1 μg/ml puromycin.

### Real-time quantitative PCR

Total RNA was extracted from cells using Trizol reagent (Yeasen, Cat#19201ES60) according to the manufacturer’s protocol. Reverse transcription was performed using cDNA Reverse Transcription Kits (Yeasen, Cat#11120ES60) according to the manufacturer’s protocols. Real-time quantitative PCR was performed using SYBR® Green Real-time PCR Master Mix (Yeasen, Cat#11201ES08). All primers used in this study are shown in Table S1.

### CCK-8 assay

Cells were seeded into 96-well plates at a density of 1 × 10^4^ cells/well and incubated at 37 °C. For NAC treatment, U87MG or U251 cells were treated with vehicle or 5 mM *N*-acetyl-l-cysteine (Sigma, Cat# A8199) for 48 h. For PRT4165 treatment, cells were treated with vehicle or 10 μM PRT4165 (MCE, Cat# HY-19817) for 48 h. For MDL29951 treatment, cells were treated with vehicle or 300 μM MDL29951 (MCE, Cat# HY-16312) for 48 h. Ten microliters of Cell Counting Kit-8 (MCE, Cat# HY-K0301) was then added to each well and the cells were incubated for 2 h at 37 °C. The OD_450_ was measured by a microplate reader (Tecan).

### BrdU immunofluorescence assay

Cells were grown on coverslips in the 24-well plates (1 × 10^4^ cells/well) overnight, and then incubated with BrdU (10 μg/ml) for 1 h. Cells were then washed with PBS, fixed in 4% paraformaldehyde for 15 min, and permeabilized with 2 M HCl at 37 °C followed by 0.1 M sodium tetraborate. The cells were blocked for 30 min, incubated with a primary antibody against BrdU for 3 h, and then incubated with the secondary antibody Alexa Fluor 555 for 2 h. Incubation with 1 μM DAPI for 20 min was used for counterstaining.

### Determination of apoptotic cells

Cells were plated onto a six-well plate at 5 × 10^5^/well and cultured for 24 h. The cells were harvested with trypsinization and were washed with 1 ml ice-cold PBS/well and resuspended in 300 μl binding buffer/well. Then, 5 μl of annexin V (ThermoFisher, Cat# V13245) conjugated with FITC and 2 μl of propidium iodide (ThermoFisher, Cat# V13245) solutions were added and mixed with the resuspended cells. After 15 min incubation in dark on ice, the cells were analyzed by flow cytometry.

### Mitochondrial superoxide (MitoSOX) assays

Cells were treated with Superoxide-reacting dye MitoSOX Red (ThermoFisher, Cat# M36008) and subjected to flow cytometry, following the manufacturer’s instruction.

### Dual-luciferase reporter assay

The 293T, U87MG, U251 cells were seeded into 24-well plates and cultured to 80–90% before transfection. Then the cells were transfected with the indicated reporter plasmids. After 48 h, firefly and Renilla luciferase activities were detected by Dual-luciferase Reporter Assay System (Promega, Cat# E1960) following the manufacturer’s instruction.

### Intracellular cAMP concentration assessment

To assess intracellular cAMP concentration, cells were cultured in 96-well plates at a density of 1 × 10^4^/well. The cAMP level was quantified using the cAMP-Glo Assay Kit (Promega, Cat# V1501) following the manufacturer’s instructions.

### Hematoxylin eosin (H&E) staining

After deparaffinization and rehydration, 5 μm sections were stained with hematoxylin solution (Sigma, Cat# HHS16) for 8 min and rinsed in distilled water for 10 min. Then the sections were stained with eosin solution (Sigma, Cat# HT110316) for 2 min and followed by dehydration with graded alcohol and cleared in xylene. The mounted slides were then examined and photographed.

### Immunohistochemistry (IHC) staining

Human glioma and normal tissues chip (Cat.#HBraG090PG01) were obtained from Shanghai Outdo Biotech. Co. Ltd (Shanghai, China). Subcutaneous xenotransplanted tumors were fixed in 4% PFA and embedded in paraffin. IHC staining was performed according to the manufacturer’s instructions (MXB, Cat.#KIT-9720). The stainings were evaluated blindly by two independent observers. The IHC score was calculated by multiplying the staining grade (+0 unstained, +1 weak, +2 moderate, and +3 strong) with the staining ratio of cells (+0 <5%, +1 5–25%, +2 26–50%, +3 51–75%, and +4 >75%). A score <3 was negative, while a score ≥3 was positive.

### ChIP-Seq and ChIP-qPCR

RNF2 or H2AK119ub ChIP assays were performed in U87-shScr/U87-shGPR17, U87-Vec/U87-GPR17 stable cells, or wild-type U87MG cells treated with Vehicle/MDL29951 (300 μM) for 48 h. In brief, approximately 5 × 10^6^ cells were cross-linked with 1% formaldehyde for 15 min at room temperature and quenched with 140 mM glycine for 10 min. Chromatin was cross-linked and sonicated to obtain fragments between 200 and 400 bp. Chromatin was used for immunoprecipitation by incubation with RNF2 antibody (5 μg) or H2AK119ub antibody (5 μg) overnight at 4 °C. Immunoprecipitated complexes were collected using 70 μl protein A/G Magnetic Beads (MCE, Cat# HY-K0202). Subsequently, beads were washed, and then eluted in 300 μl of elution buffer overnight at 65 °C. After reverse crosslinking, DNA was then purified on columns. The CHIP-Seq DNA libraries were sequenced on the Illumina sequencing platform Hi-Seq X-Ten by Amogene Biotechnology Co., Ltd (Xiamen, China).

### ChIP-Seq analysis

Fastq data were processed with Trimmomatic v0.39 to remove low-quality reads and then reads of ChIP-seq data were aligned to the hg19 genome using Bowtie v2.3.5. Peak calling was performed using MACS2 (V2.1.2) (Model-based Analysis of ChIP-Seq) (http://liulab.dfci.harvard.edu/MACS) with a *p* value cutoff of 0.01. For the differential binding peaks of RNF2 ChIP-seq, MAnorm (V1.3.0) was used with cutoff *p* < 0.05 and fold change >2. ChIP-seq files were converted to BigWig files using deepTools 3.1.3 and visualized by IGV 2.4.16.

### RNA-Seq analysis

RNA-Seq analysis with U87-Vec and U87-GPR17 cells were performed by Novogene (Beijing, China). All RNA-Seq data were aligned to hg19 by Hisat2 v2.0.5. FeatureCounts v1.5.0-p3 and StringTie(v1.3.3b) were used to generate gene counts. Differential expressed genes were identified using DESeq2 with fold change >1.5 and *p* < 0.05. GO-analysis of differentially expressed genes was performed with DAVID (https://david.ncifcrf.gov/).

### TCGA and CGGA data preparation and analysis

RNA-seq raw counts of 511 low-grade glioma (LGG) and 156 glioblastoma (GBM) cases from The Cancer Genome Atlas (TCGA) cohort were obtained from the Genomic Data Commons (GDC) data portal. Differentially expressed genes were identified using DESeq2 with fold change >2 and *P*-adj <0.05. GSEA was performed with GSEA v4.1.0 for all differentially expressed genes.

Primary tumor expression data of 282 LGG and 140 glioblastoma (GBM) cases were obtained from the Chinese Glioma Genome Atlas (CGGA) database(mRNAseq_693).Differentially expressed genes were identified using the limma-voom tool with fold change >2 and *P*adj < 0.05.

### Xenograft mouse model

For the subcutaneous model, 14 male BALB/c nude mice (age, 5–6 weeks) were randomly divided into two groups, with seven mice in each group. 1 × 10^7^ U87 cells were subcutaneously injected, and the tumor size was measured with the caliper and calculated by the formula *V* = [1/2] *ab*^2^ (*a* and *b* present the long and short diameters of the tumor, respectively).

For the intracranial model, a total of 10 male BALB/c nude mice (age, 5–6 weeks) were randomly divided into two groups, with five mice in each group. 5 × 10^5^ U87-Vec and U87-GPR17 cells were injected at 2 mm anterior and 1.5 mm lateral of the right hemisphere relative to bregma at a depth of 3 mm of the nude mice with the stereotaxic instrument. Data acquisition and analysis was performed using the Living Image® software (Caliper LS). Tumor samples were collected after imaging.

All mice were obtained from the Experimental Animal Center (Xiamen University,China). All animal experiments conformed to ethical principles and guidelines approved by Xiamen University Animal Care and Use Committee.

### Antibodies

Cleaved-caspase3 (Affinity, Cat# AF7022), Caspase3 (CST, Cat# 9662), RNF2 (Abcam, Cat# ab181140), H2A (CST, Cat# 12349), H2AK119ub (CST, Cat# 8240), H3 (Abcam, Cat# ab1791), H3K27me3 (Abcam, Cat# ab6002), H3K27Ac (CST, Cat# 4353), H3K9me1 (Abcam, Cat# ab8896), H3K4me1 (Abcam, Cat# ab8895), Phosphorylated-P65 (CST, Cat# 3033), P65 (CST, Cat# 8242), PKA (CST, Cat# 4782), P-PKA (Santa Cruz, Cat# sc-32968), GAPDH (Proteintech, Cat# 60004–1-lg), BrdU (Abcam, Cat# ab6326), Ki67 (GeneTex, Cat# GTX16667).

### Western blot analysis

The cells were lysed in cell lysis buffer (20 mM Tris-HCl, pH 7.5, 150 mM NaCl, 1 mM EDTA, 1 mM EGTA, 1% Triton X-100, 2.5 mM sodium pyrophosphate, 1 mM β-glycerophosphate, 1 mM NaF, 1 mM PMSF), supplemented with protease inhibitor cocktail (MCE, Cat# HY-K0010), phosphatase inhibitor cocktail I (MCE, Cat# HY-K0021), and phosphatase inhibitor cocktail II (MCE, Cat# HY-K0022) on ice for 30 min followed by centrifugation at 12,000 r.p.m. for 30 min. Protein concentrations were measured using the BCA Protein Assay Kit (Sangon Biotech, Cat# C503021). Proteins were separated on SDS-PAGE and the protein marker (Yeasen, Cat# 20351ES72) was used to indicate the size of proteins.

### Statistical analysis

The statistical analyses were performed with Graphpad Prism 5. The data for two-group comparisons were analyzed for statistical significance using two-tailed Student’s *t*-tests. Normal distribution and similar variances were assumed. Error bars represented standard error of measurement (s.e.m.). *P* values were indicated with single asterisk (* < 0.05), double asterisks (** < 0.01), and triple asterisks (*** < 0.001) on graphs. The number for each experiment was stated in figure legends.

## Results

### GPR17 suppressed glioma tumorigenesis

**Fig. 1:**
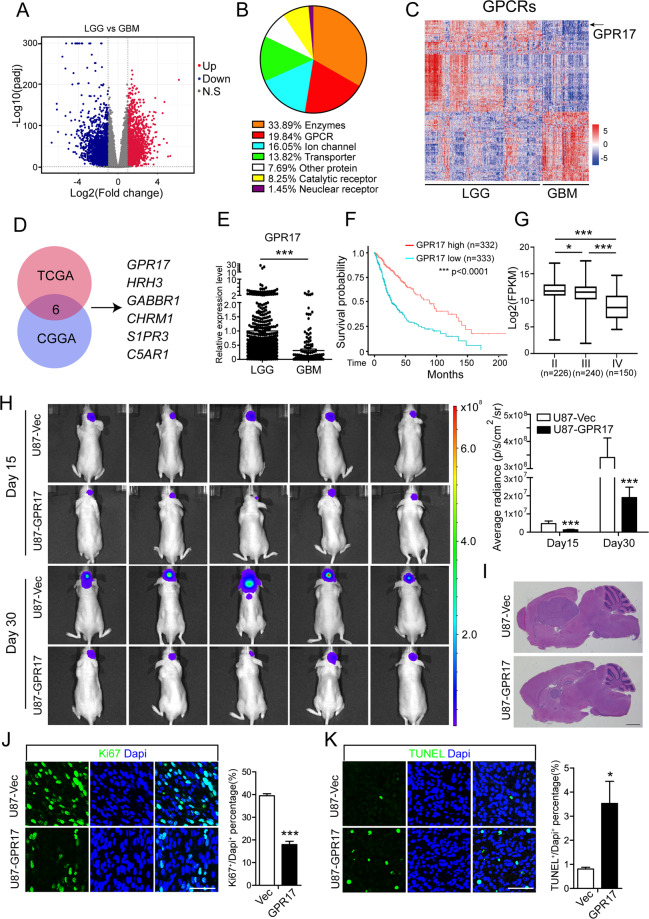
GPR17 suppressed glioma tumorigenesis. **A** Volcano plot of the differentially expressed protein-coding genes between LGG (*n* = 511) and GBM (*n* = 156) from the TCGA cohort. **B** Pie chart showing the proportions of the drug targets in differentially expressed genes. **C** Heatmap analysis of the differentially expressed GPCRs between LGG and GBM patients from TCGA dataset. Gene expression values were *z*-transformed and were colored red for high expression and blue for low expression. Black arrow indicated GPR17. **D** Venn diagram showing the differentially expressed GPCRs found in both TCGA and CGGA database after screening. **E** mRNA levels of *GPR17* in LGG (*n* = 511) and GBM (*n* = 156) patients from the TCGA cohort. **F** Kaplan–Meier analysis of patients overall survival data based on high versus low expression of *GPR17* in glioma, grades II–IV, from the TCGA dataset. *P* values were obtained from the log-rank test. ***p < 0.001, **G** Expression of *GPR17* in grade II (*n* = 226), grade III (*n* = 240), and GBM (*n* = 150) patients from the TCGA cohort. **H** BALB/c nude mice (*n* = 5 per group) were xenotransplanted with U87-Vec or *GPR17*-overexpressing U87MG cells through stereotatic injection. Bioluminescence imaging was performed on days 15 and 30 after xenotransplantation. Data represent the means ± SEM from five mice. ***P < 0.001, Student’s *t-*test. **I** H&E staining of brain sections with U87-Vec and U87-GPR17 tumors. Scale bar, 300 μm. **J**, **K** Immunofluorescent staining against Ki67 (J) and TUNEL (**K**) of the tumor sections. Scale bar, 50 μm.

At present, the mechanism underlying the development of glioma is still a most popular topic in tumor research. To demonstrate the transcriptomic difference between LGG and GBM, we performed data mining using the RNA-Seq data from the tumor samples in 511 LGG and 156 GBM patients in TCGA database (https://www.cancer.gov/tcga). Compared with LGG samples, 2261 genes were upregulated and 1974 genes were downregulated in GBM samples (Fig. 1A). Gene set enrichment analysis (GSEA) revealed that they were involved in multiple pathways including adenylate cyclase inhibiting GPCR signaling and regulation of cAMP-mediated signaling pathways (Fig. S1). According to the functions of the proteins, over 19% of differentially expressed drug targets were G protein-coupled receptors (Fig. 1B). In GBM samples, 78 GPCRs were upregulated and 100 GPCRs were downregulated (Fig. 1C).

A similar analysis was performed with the patient data from the Chinese Glioma Genome Atlas (CGGA) [21, 22] (https://cgga.org.cn/). When we combined both analyses, we found six GPCRs with established small-molecule ligands, which might facilitate their potential application for glioma therapy. So far, the roles of these 6 GPCRs in glioma were poorly studied. Among them, *GPR17* was one of genes with the most significant change in their expression levels between LGG and GBM patients (Fig. 1D). In the tumor samples from GBM patients, the expression of *GPR17* was about 32% of the level in LGG samples (Fig. 1D). Previously, we found that GPR17 promoted cell death in oligodendrocyte by regulating the expression of a pro-apoptotic gene *Xaf1* (ref. [16]). As some glioma originated from oligodendrocyte lineage cells [23, 24], we speculated that GPR17 played a role in glioma development. Until now, although several studies have mentioned that GPR17 was involved in glioma [18–20], the functions of GPR17 during glioma development and the underlying mechanism were largely unstudied.

In TCGA database, patients with high expression of *GPR17* survived longer than the patients with a low level of GPR17 (Fig. 1F), and the Gliovis database (http://gliovis.bioinfo.cnio.es/) revealed that the expression level of *GPR17* gradually reduced during glioma exacerbation from Grade II to Grade IV (Fig. 1G). These data clearly suggested that the expression of *GPR17* is negatively correlated with glioma development.

To directly test the potential role of GPR17 in glioma development, we generated a stable *GPR17*-overexpressing cell line with a common used glioma cell line, U87MG (U87-GPR17) [25–27] (Fig. S2). We then transduced both control and U87-GPR17 cells with a luciferase-encoding vector using a Lenti-virus system and transplanted them into cerebral cortex. On days 15 and 30 posttransplantation, we carried out luminescent imaging and observed a dramatically and consistently suppressed tumor formation in the BALB/c nude mice with U87-GPR17 cells, compared to the mice injected with U87-Vec cells (Fig. 1H). Furthermore, hematoxylin eosin (H&E) staining confirmed the suppressed tumor formation by *GPR17* overexpression (Fig. 1I). Immunofluorescent imaging displayed that the cells in *GPR17*-overexpressing tumors had a lower degree of proliferation and a higher degree of cell death, as shown by the reduced Ki67+ and increased TUNEL+ cell population (Fig. 1J, K).

### GPR17 suppressed glioma cell proliferation and induced apoptotic death through augmenting reactive oxygen species (ROS) level

**Fig. 2:**
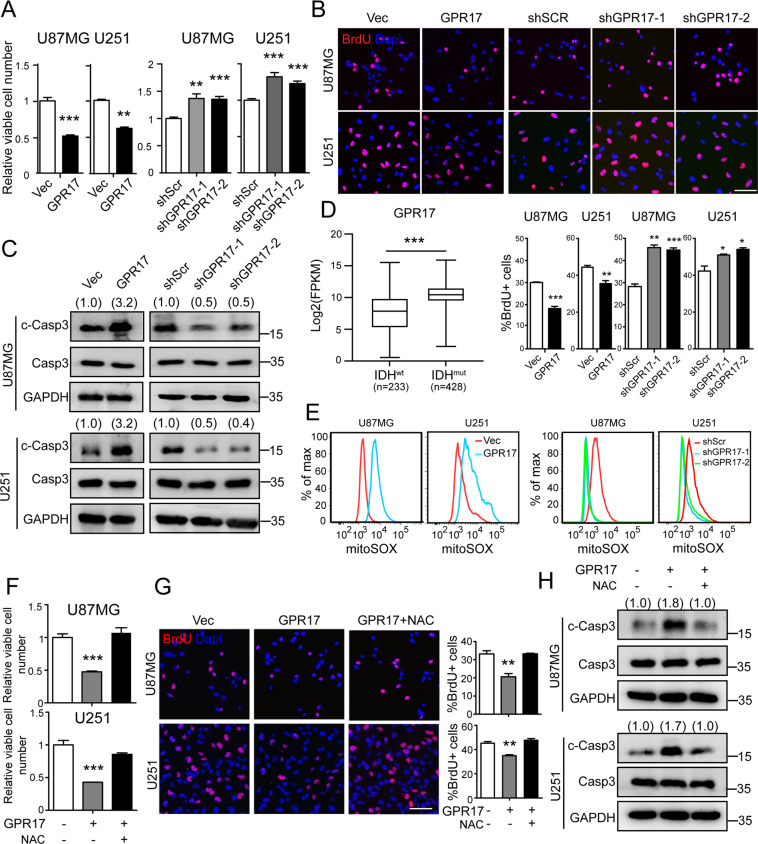
GPR17 inhibited cell proliferation and induced cell death by inducing ROS level. GPR17-overexpressing/knockdown U87MG or U251 stable cell lines (U87/U251-GPR17 or U87/U251-shGPR17) were generated as described in “Materials and methods”. After confirming the overexpression or knockdown efficiencies of GPR17, cells were cultured and harvested for the subsequent experiments. **A** CCK-8 assay was performed to examine viable cell numbers. **B** Immunofluorescent staining against BrdU was performed to examine the numbers of proliferating cells, scale bar, 400 μm. The proportions of the BrdU+ cell in the total cell number were displayed in the lower panel. **C** Western blot was performed to examine intracellular cleaved-caspase3 levels. Densitometric quantification of cleaved-caspase3/caspase3 ratio from at least three independent assays was indicated on top of each band, respectively. **D** Expression of *GPR17* in IDH^wt^ (*n* = 233) and IDH^mut^ (*n* = 428) glioma patients from the TCGA cohort. **E** Flow cytometry analysis was performed to assess mitochondrial ROS level. **F**–**H** Control and U87/U251-GPR17 cells were treated with vehicle or *N*-acetyl-l-cysteine (5 mM) for 48 h, CCK-8 assays (**F**), immunofluorescent staining against BrdU (**G**), and western blotting against cleaved-caspase3 (**H**) were performed as mentioned above. Scale bar, 400 μm. Densitometric quantification of cleaved-caspase3/caspase3 ratio from at least three independent assays was indicated on top of each band, respectively. For all panels, data represent the means ± SEM from three independent experiments. *p < 0.05, **p < 0.01, ***p < 0.001, Student’s *t*-test.

To demonstrate the molecular basis of GPR17 in glioma development, we stably overexpressed or knocked down *GPR17* in two glioma cell lines, U87MG (U87) and U251 (Fig. S2). Overexpression of *GPR17* reduced their cell viability, while knockdown of *GPR17* increased viable cell numbers (Fig. 2A). Immunofluorescent imaging against BrdU indicated that GPR17 inhibited glioma cell proliferation (Fig. 2B). Furthermore, we found that overexpression of *GPR17* induced apoptosis, and knockdown of *GPR17* protected cells from apoptotic death (Figs. 2C and S3). However, GPR17 had no effect on glioma cell invasion (Fig. S4).

Previously, GPR17 was characterized as a prognostic signature for glioblastoma patients with wild-type IDH [20]. Further, in TCGA database, we found that glioma patients with IDH mutations showed higher expression level of GPR17 than the patients with wild-type IDH (Fig. 2D). As reported, IDH mutation in glioma cells was associated with the increased intracellular ROS level [28, 29]. We therefore examined the ROS levels in our GPR17-overexpressing and silencing glioma cells, aiming to reveal the molecular basis leading to the suppressed cell proliferation and survival by GPR17. Figure 2E showed that overexpression of GPR17 increased the intracellular ROS level, whereas knockdown of GPR17 reduced the ROS level in U87MG or U251 cells. These results suggested that the inhibitory effects of GPR17 on glioma cell growth and survival were mediated by oxidative stress. To confirm the hypothesis, we used *N*-acetyl cysteine (NAC) to eliminate ROS [30, 31]. NAC treatment rescued the reduced viable cell population by *GPR17* overexpression, through promoting cell proliferation and inhibiting apoptosis (Fig. 2F–H). These data indicated that the inhibitory effect of GPR17 on glioma development resulted from the reductive effect of GPR17 on glioma cell viability, through its regulation on oxidative stress.

### GPR17 reduced histone monoubiquitination controlled by RNF2 in glioma cells

**Fig. 3:**
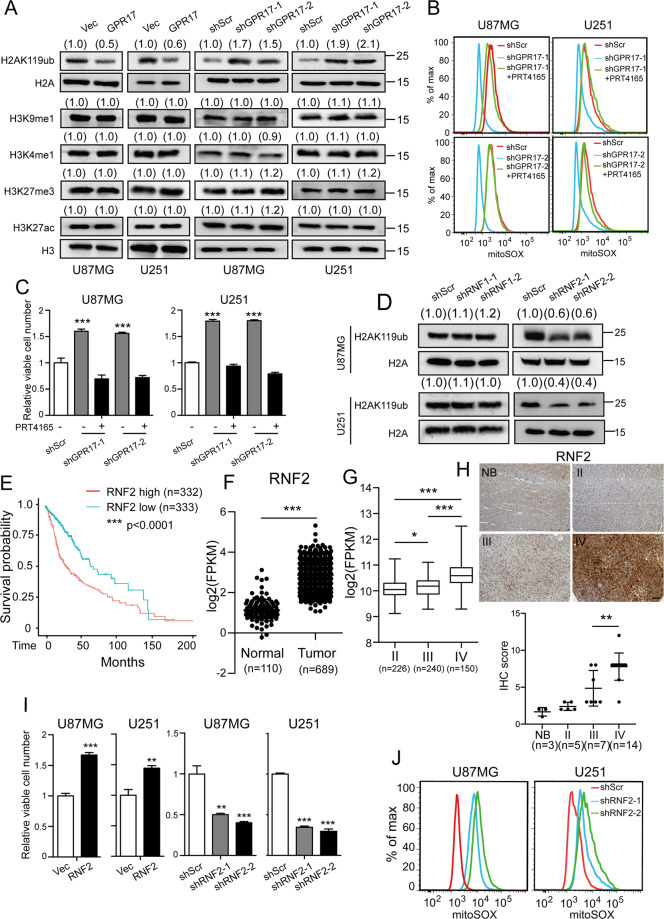
GPR17 inhibited RNF2-mediated histone monoubiquitination. U87/U251-GPR17 or U87/U251-shGPR17 cells were prepared as described in Fig. 2. **A** Western blot was performed to detect the indicated protein levels. Densitometric quantification of H2AK119ub/H2A, H3K9me1/H3, H3K4me1/H3, H3K27me3/H3, or H3K27ac/H3 ratio from at least three independent assays was indicated on top of each band, respectively. **B**, **C** Control and U87/U251-shGPR17 cells were treated with/without 10 μM PRT4165 for 48 h. Flow cytometry analysis was performed to assess mitochondrial ROS level (**b**), and CCK-8 assay was performed to examine viable cell numbers (**C**). **D** Control (Scramble), RNF1, or RNF2 shRNAs were transfected to U87MG or U251 cells to knockdown their expressions. Cells were then subjected to western blot assays to test the H2AK119ub level. Densitometric quantification of H2AK119ub/H2A ratio from at least three independent assays was indicated on top of each band, respectively. **E** Kaplan–Meier analysis of patients overall survival data based on high versus low expression of RNF2 in gliomas, grades II–IV, from the TCGA dataset. **F** Expression of RNF2 in normal (*n* = 110) and tumor (*n* = 689) from the TCGA cohort. ***p < 0.001, Student’s *t-*test. **G** Expression of RNF2 in grade II (*n* = 226), grade III (*n* = 240), and GBM (*n* = 150) patients from the TCGA cohort. **H** RNF2 expression in normal brain (NB) tissues or glioma was examined by IHC staining (NB, *n* = 3; Grade II, *n* = 5; Grade III, *n* = 7, Grade IV, *n* = 14), scale bar 250 μm. **p < 0.01, Student’s *t-*test. **I**, **J** RNF2 overexpressing/knockdown U87MG or U251 stable cell lines (U87/U251-RNF2 or U87/U251-shRNF2) were generated as described in “Materials and methods”. After confirming the overexpression or knockdown efficiencies of *RNF2*, cells were cultured and harvested for CCK-8 assays to examine viable cell numbers (**I**), or flow cytometry analysis to assess mitochondrial ROS level (**J**).

We have previously shown that GPR17 regulated transcription [16, 17], but whether and how GPR17 was involved in gene regulation in tumor cells remained unknown. Epigenetic modification on histone controlled chromatin structure and functions, and it played an important role in the regulation of gene expression [32, 33]. In our study, we observed that overexpression of GPR17 decreased the intracellular H2AK119ub level. Furthermore, knocking down GPR17 showed inductive effect on H2AK119ub level, suggesting that GPR17 was involved in histone monoubiquitination, which was tightly connected with tumorigenesis [34–37], in both U87MG and U251 cells (Fig. 3A). Meanwhile, overexpression or knockdown of *GPR17* had no effect on H3K9me1, H3K4me1, H3K27me3, or H3K27ac levels (Fig. 3A), indicating that GPR17 did not affect histone methylation or acetylation. Previous studies showed that Polycomb repressive complex 1 (PRC1) primarily catalyzed the monoubiquitination of the lysine 119 in histone H2A subunit [38, 39]. We therefore used PRT4165, a PRC1 complex inhibitor [40, 41], to inhibit PRC1 complex activity, aiming to test whether GPR17 functioned through PRC1 complex. Knockdown of *GPR17* reduced the ROS level (Fig. 3B) and increased cell viability (Fig. 3C). When the GPR17-silencing cells were treated with PRT4165, the ROS levels or the viable cell numbers returned to similar levels as those in U87/U251-shScr cells, suggesting that the inhibitory effect of GPR17 on glioma development was mediated by PRC1-mediated histone monoubiquitination.

In the PRC1 complex, RNF1 and RNF2 were the key components to regulate histone monoubiquitination [38, 39]. We then knocked them down in U87MG and U251 cells, to find out which of them was mainly responsible for histone monoubiquitination in glioma cells. As shown in Fig. 3D, knockdown of *RNF2*, but not *RNF1*, reduced the H2AK119ub level, indicating that RNF2 was the main factor for histone monoubiquitination in glioma cells.

Previously, multiple studies have shown that RNF2 functioned widely in tumorigenesis [36, 42]. We then explored the role of RNF2 in glioma development. In TCGA database, patients with high *RNF2* expression survived shorter than the patients with low *RNF2* expression (Fig. 3E). The Gliovis database showed that in tumor tissues, the expression of *RNF2* was significantly higher than normal tissues (Fig. 3F), and *RNF2* expression increased during glioma exacerbation, from Grade II to IV (Fig. 3G). We also confirmed the protein levels of RNF2 in a cohort of glioma and normal brain tissue samples. The level of RNF2 was relatively higher in high-grade gliomas (WHO IV) than in normal brain tissues or LGGs (WHO II and III) (Fig. 3H). Thus, these data indicated that RNF2 might promote the tumorigenesis of glioma. To investigate its biological functions, we then generated *RNF2* overexpressing or silencing stable cell lines for further study (Fig. S5). Figure 3I showed overexpression of *RNF2* augmented viable cell population in both U87MG and U251 cells, whereas knocking-down *RNF2* reduced viable cell numbers. RNF2 also promoted glioma cell proliferation and inhibited cell apoptosis (Fig. S6). And knockdown of *RNF2* effectively elevated intracellular ROS levels (Fig. 3J). These data clearly revealed that RNF2 functioned oppositely to GPR17.

### GPR17 suppressed *RNF2* expression through a cAMP/PKA/p65 signaling pathway

**Fig. 4:**
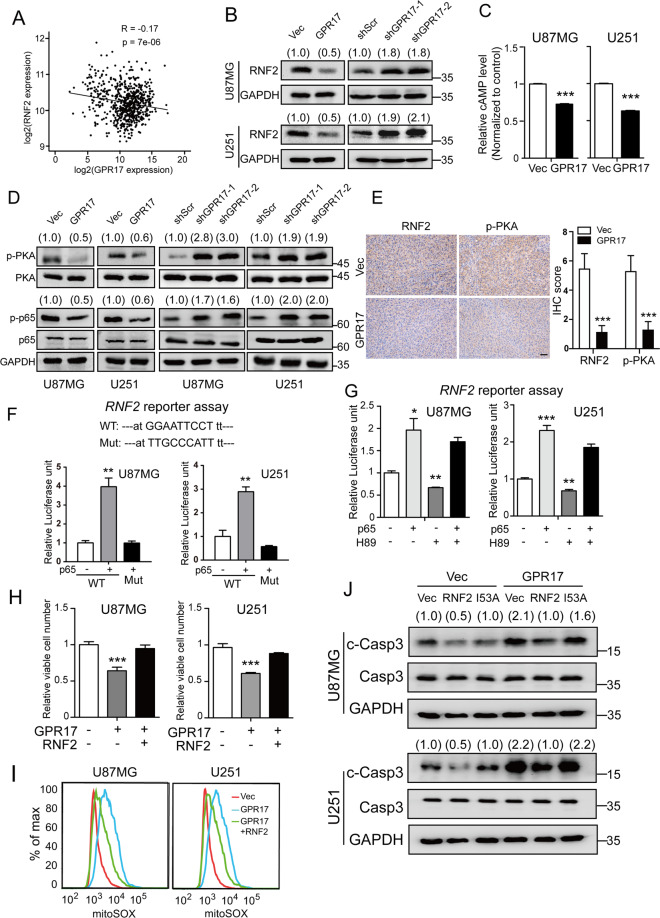
GPR17 suppressed the expression of RNF2 through cAMP/PKA/p65 axis. **A** Correlation plot of the mRNA levels of GPR17 and RNF2 in glioma from 667 patient samples in TCGA dataset. **B** Control, U87/U251-GPR17, or U87/U251-shGPR17 cells were subjected to western blot to assess the protein level of RNF2. **C** cAMP levels were measured in control and U87/U251-GPR17 cells. **D** Control, U87/U251-GPR17, or U87/U251-shGPR17 cells were subjected to western blot to detect the indicated protein levels. Densitometric quantification of p-PKA/PKA, or p-p65/p65 ratio from at least three independent assays was indicated on top of each band, respectively. **E** The protein levels of RNF2 or p-PKA of brain sections with U87-Vec and U87-GPR17 tumors were evaluated by IHC analysis. Scale bar 50 μm. **F**, **G** U87MG or U251 cells were co-transfected with wild type (WT) or mutant RNF2 promoter (2.0 kb)-firefly luciferase, Renilla luciferase, and p65-overexpressing vectors for 24 h (**F**); or co-transfected with WT RNF2 promoter (2.0 kb)-firefly luciferase, Renilla luciferase and p65-overexpressing vectors for 24 h, and then treated with or without 10 μM H89 treatment for 48 h (**G**). Cells were then harvested for dual-luciferase reporter assay following the manufacturer’s instructions. **H**, **I** Control or U87/U251-GPR17 cells were transfected with control or RNF2-overexpressing vectors for 48 h, and then CCK-8 assays were performed to measure viable cell numbers (**H**), flow cytometry analyses were performed to assess mitochondrial ROS levels (**I**). **J** Control or U87/U251-GPR17 cells were transfected with RNF2 wild type/I53A mutant-overexpressing vectors for 48 h. Cells were then harvested for western blot against cleaved-caspase3. Densitometric quantification of cleaved-caspase3/caspase3 ratio from at least three independent assays was indicated on top of each band, respectively. For all panels, data represent the means ± SEM from three independent experiments. *p < 0.05, **p < 0.01, ***p < 0.001, Student’s *t-*test.

So far, there is no study depicting the relationship between GPR17 and RNF2. Here, we found in TCGA database that the expression levels of *GPR17* and *RNF2* were negatively correlated, suggesting that GPR17 could negatively regulate the expression of *RNF2* (Fig. 4A). To test the hypothesis, we overexpressed *GPR17* in U87MG and U251 cells, and found that *RNF2* expression was significantly reduced, whereas knocking down *GPR17* showed inductive effects (Fig. 4B).

Previous studies showed that the expression of *RNF2* was regulated by NF-κB signaling [43], and the activity of p65, the key factor in NF-κB signaling, was regulated by PKA signaling activity [44, 45]. We then hypothesized that GPR17 regulated *RNF2* gene expression through NF-κB pathway and performed experiments to confirm our hypothesis. In both U87MG and U251 cells, we observed that overexpressing *GPR17* reduced cAMP level (Fig. 4C), and suppressed PKA and NF-κB signaling (Fig. 4D). On the other hand, knockdown of *GPR17* activated PKA signaling and induced the p-p65 level (Fig. 4D). In addition, relatively lower RNF2 and p-PKA protein level were also consistently detected in intracranial U87-GPR17 xenograft tumors (Fig. 4E). We then performed dual-luciferase reporter assay, and found that p65 directly interacted with *RNF2* promoter, and then induced *RNF2* gene expression (Fig. 4F). When the p65 response element in the *RNF2* promoter was mutated, the regulatory effect of p65 was abolished (Fig. 4F). Furthermore, inhibition on PKA signaling by its inhibitor H89 reduced the mRNA transcription level controlled by *RNF2* promoter, but overexpression of p65 rescued the transcription level (Fig. 4G).

Moreover, we observed that RNF2 reversed the inhibitory effect of GPR17 on cell viability (Fig. 4H). Overexpression of RNF2 sufficiently restored the increased ROS and the cleaved-caspase3 level by GPR17 overexpression (Fig. 4I, J). When the Ile53, the key residue for the monoubiquitination catalyzing function of RNF2, was mutated to alanine, the mutant could no longer repress the increased cleaved-caspase3 level by GPR17 (Fig. 4J). Taken together, our results indicated that GPR17 regulated *RNF2* expression through a cAMP–PKA–p65 regulatory axis, and RNF2 mediated the functions of GPR17 in glioma cells.

### *KLF9* was a downstream target for GPR17 and RNF2 in glioma cells

**Fig. 5:**
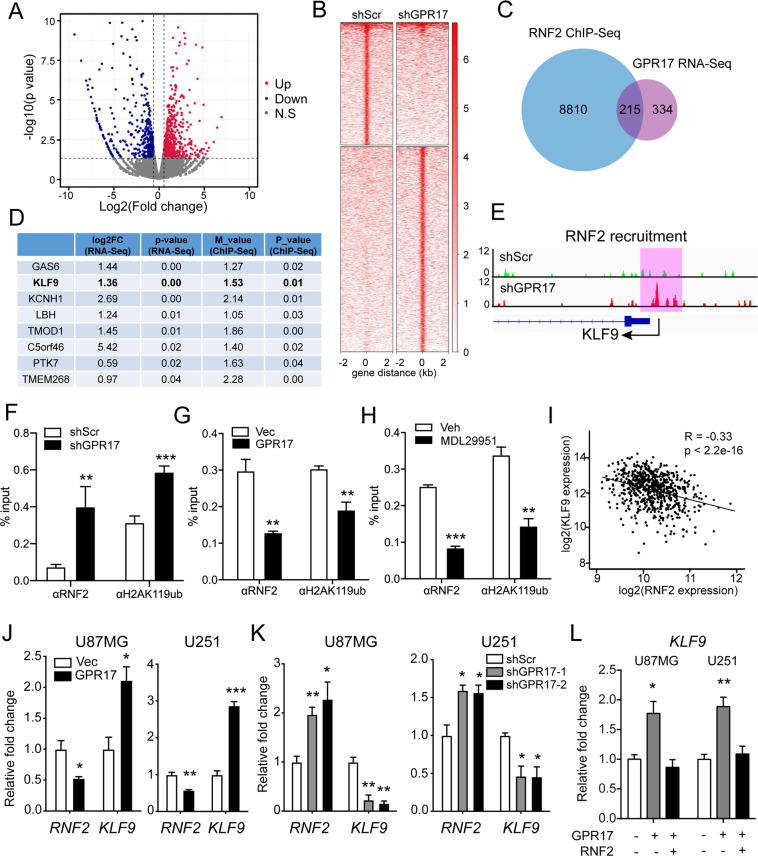
KLF9 was a downstream target for GPR17 and RNF2 in glioma cells. **A** Control and U87-GPR17 cells were subjected to RNA-Seq analysis. Volcano plot depicted gene expression changes between control and U87-GPR17 cells. **B** Control and U87-shGPR17 cells were subjected to RNF2 ChIP-Seq analysis. Density heatmap showed the RNF2 recruitment within ±2 kb around the RNF2 peak center. **C** Venn diagram showing the overlapped genes in ChIP-Seq and RNA-Seq analyses. **D** Gene list ranked by RNA-Seq *p* value with RNF2 binding on promoter-TSS region. **E** Diagram showing RNF2 enrichment on *KLF9* promoter-TSS region. **F**–**H** U87-shScr/shGPR17 (**f**), U87-Vec/GPR17 (**G**), or U87MG cells treated with Vehicle/MDL29951 (300 μM) for 48 h (**H**) were subjected to RNF2 and H2AK119ub ChIP assay; real-time PCR analysis was then performed to assess the relative enrichment of RNF2 and H2AK119ub in promoter-TSS region of *KLF9*. **I** Correlation plot of *RNF2* and *KLF9* mRNA expression in glioma, grades II–IV, from the TCGA dataset (*n* = 667). **J**, **K** Control, U87/U251-GPR17, or U87/U251-shGPR17 cells were prepared as in Fig. 2. Cells were harvested for real-time PCR analysis to assess the mRNA levels of *RNF2* and *KLF9*. **L** Control or U87/U251-GPR17 cells were transfected with control or RNF2-overexpressing vectors for 48 h, and then subjected to real-time PCR analysis to assess the mRNA levels of *KLF9*. For all panels, data represent the means ± SEM from three independent experiments. *p < 0.05, **p < 0.01, ***p < 0.001, Student’s *t-*test.

As RNF2-containing PRC1 complex bound to chromatin and regulated gene expression by histone monoubiquitination, we attempted to identify the downstream targets for both GPR17 and RNF2. Thus, we performed RNA-Seq analysis using U87-Vec and U87-GPR17 cells. Compared to control cells, 549 genes were upregulated at least by 1.5-folds and 462 genes were downregulated to at least 66% (Fig. 5A) in U87-GPR17 cells. The significantly upregulated genes could be categorized into several biological processes including positive regulation of apoptotic process, and negative regulation of cell proliferation. And the downregulated genes were mainly involved in the biological processes, including positive regulation of cell proliferation and positive regulation of NF-κB transcription factor activity, according to Gene Ontology (GO) analysis (Fig. S7). These data were consistent with our findings shown above.

We then performed RNF2 ChIP-Seq analysis to study the recruitment of RNF2 in the genome using U87-shScr or U87-shGPR17 cells (Fig. 5B). Overall, RNF2 recruited differently in 9025 genes throughout the genome, and the expressions of 215 genes were significantly downregulated in the RNA-Seq analysis (Fig. 5C).

To identify the genes mediating the effects of GPR17 and RNF2 on ROS level, we combined both analyses and screened for the genes with most significant changes in gene expression (RNA-Seq) and in RNF2 recruitment (*M* value, ChIP-Seq) in the promoter region (Fig. 5D). We then performed real-time PCR to further confirm the regulation of these target genes by GPR17 and RNF2. As shown, the expression of *KLF9* was affected by both *GPR17* overexpression and PRT4165 treatment (Fig. S8). Therefore, we chose KLF9 as our candidate. Knockdown of *GPR17* induced the recruitment of RNF2 onto the *KLF9* promoter (Fig. 5E, F), and subsequently promoted H2AK119ub recruitment (Fig. 5F). On the other hand, both RNF2 and H2AK119ub recruitments were reduced upon *GPR17* overexpression or activation by MDL29951 treatment in glioma cells (Fig. 5G, H). In the TCGA database, we observed that the expression patterns of *RNF2* and *KLF9* were negatively correlated to each other (Fig. 5I). Overexpression of *GPR17* simultaneously reduced the expression of *RNF2* but induced the expression of *KLF9*, whereas knockdown of *GPR17* had totally opposite effects (Figs. 4B and 5J, K). Moreover, overexpression of *GPR17* induced the expression level of *KLF9*, but the inductions were largely abolished by *RNF2* overexpression (Fig. 5L), further suggested that *KLF9* was the downstream target for GPR17 and RNF2. As the role of KLF9 in oxidative stress has been widely reported [46–49], we therefore chose KLF9 as a downstream target for GPR17 and RNF2 to control ROS level in glioma cells.

### KLF9 mediated the functions of GPR17 on ROS in glioma cells

**Fig. 6:**
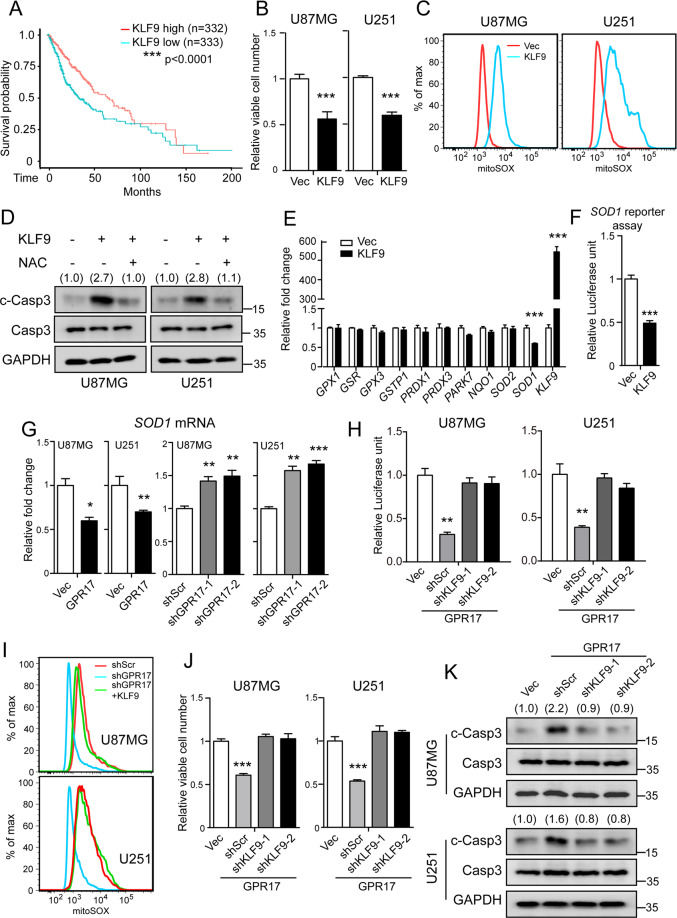
KLF9 mediated the functions of GPR17 on ROS in glioma cells. **A** Kaplan–Meier analysis of patients overall survival data based on high versus low expression of KLF9 in glioma, grades II–IV, from the TCGA dataset. **B**, **C** U87MG or U251 cells were transfected with control/KLF9-overexpressing vectors for 48 h. Cells were harvested for CCK-8 assay measuring the viable cell numbers (**B**) or flow cytometry analysis to measure intracellular ROS level (**C**). **D** U87MG or U251 cells were transfected with control or KLF9-overexpressing vectors, and then harvested for western blotting after treated with vehicle or *N*-acetyl-l-cysteine (5 mM) for 48 h. Densitometric quantification of cleaved-caspase3/caspase3 ratio from at least three independent assays was indicated on top of each band, respectively. **E** Real-time PCR analysis to assess the mRNA levels of the oxidative stress-related genes in U87MG cells transfected with control or KLF9-overexpressing vectors for 48 h. **F** HEK293T cells were co-transfected with *SOD1* promoter-firefly luciferase, renilla luciferase, and *KLF9*-overexpressing vectors for 48 h. Cells were then harvested for dual-luciferase reporter assay following the manufacturer’s instructions. **G** Control, U87/U251-GPR17, or U87/U251-shGPR17 cells were prepared as in Fig. 2. Cells were harvested for real-time PCR analysis to assess the mRNA levels of *SOD1*. **H** Control or U87/U251-GPR17 cells were transfected with scramble or KLF9-knockdown vectors, together with *SOD1* promoter-firefly luciferase, Renilla luciferase constructs for 48 h. Cells were then harvested for dual-luciferase reporter assay following the manufacturer’s instructions. **I** Control or U87/U251-shGPR17 cells were transfected with control or KLF9-overexpressing vector for 48 h. Flow cytometry analysis was performed to assess mitochondrial ROS levels. **J**, **K** Control or U87/U251-GPR17 cells were transfected with scramble or *KLF9* shRNAs for 48 h. Cells were then harvested for CCK-8 assays (**J**) or western blot against cleaved-caspase3 (**K**). Densitometric quantification of the cleaved-caspase3/caspase3 ratio from at least three independent assays was indicated on top of each band, respectively. For all panels, data represent the means ± SEM from three independent experiments. *p < 0.05, **p < 0.01, ***p < 0.001, Student’s *t-*test.

KLF9 has been reported as an important regulator controlling the ROS level. KLF9 promoted the accumulation of ROS by suppressing the expressions of reductase in cells [46–49]. Therefore, we hypothesized that KLF9 might mediate the regulatory effects of GPR17 and RNF2 on ROS levels and glioma tumorigenesis. To test our hypothesis, we firstly analyzed the survival curves of the glioma patients with high or low *KLF9* expression from TCGA database, and found that patients with high *KLF9* expression survived longer than patients in the other group (Fig. 6A). In cultured glioma cells, overexpression of *KLF9* reduced viable cell numbers (Fig. 6B), elevated ROS levels (Fig. 6C), and induced intracellular cleaved-caspase3 levels (Fig. 6D). Administration of NAC effectively reduced the intracellular cleaved-caspase3 levels by KLF9 overexpression (Fig. 6D). Mechanistically, we examined the expression of the key genes controlling ROS and oxidative stress in cells [50–52], and found that KLF9 reduced the expression of *SOD1* gene (Fig. 6E), whose encoding protein played an essential role in the clearance of ROS in cells [53]. *SOD1* reporter assay displayed that KLF9 controlled *SOD1* gene expression by a direct binding to its promoter region (Fig. 6F). As *KLF9* was a downstream target of GPR17, we therefore test that whether the expression of *SOD1* was subjected to GPR17 regulation. As expected, overexpression of GPR17 reduced the expression of *SOD1*, while knockdown of GPR17 showed inductive effects, in both U87MG and U251 cells (Fig. 6G). Furthermore, *SOD1* reporter assay showed that overexpression of GPR17 reduced *SOD1* promoter activity, whereas knockdown of KLF9 rescued the promoter activity (Fig. 6H).

To test whether KLF9 mediated the biological functions of GPR17, we overexpressed *KLF9* in *GPR17*-knockdown U87MG and U251 cells, and found that overexpression of *KLF9* abolished the reduced ROS level by GPR17 silencing (Fig. 6I). In contrast, knocking down *KLF9* rescued the suppressed cell viabilities and diminished the increased intracellular cleaved-caspase3 caused by *GPR17* overexpression (Fig. 6J, K). These data suggested that KLF9 sufficiently mediated GPR17 functions in glioma cells.

### Activation of GPR17 inhibited glioma formation

**Fig. 7:**
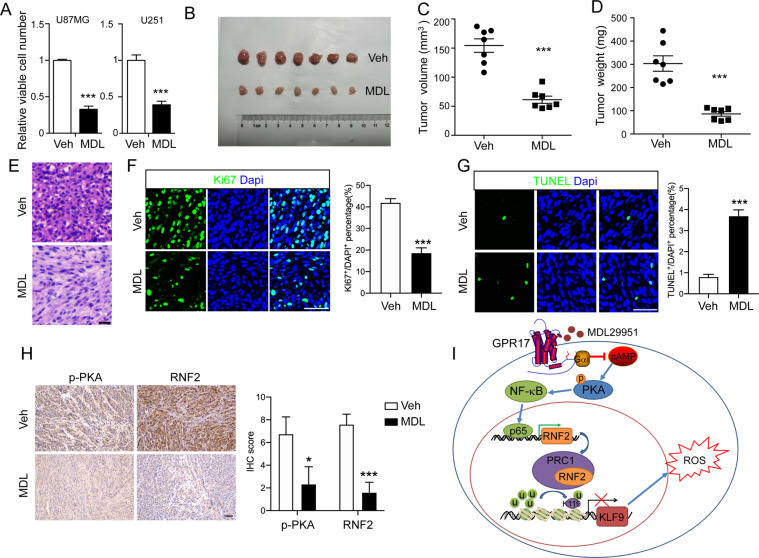
Activation of GPR17 inhibited glioma formation. **A** U87MG and U251 cells treated with vehicle or MDL29951 (300 μM) for 48 h, and CCK-8 assay was performed to examine viable cell numbers. Data represent the means ± SEM from three independent experiments. **B**–**D** BALB/c nude mice with subcutaneously xenotransplanted tumor were intraperitoneally injected with 10 mg/kg MDL29951 for 16 days, and then tumor samples were collected for the measurements of tumor sizes (**B**), volumes (**C**), and weights (**D**), (*n* = 7). **E** H&E staining of sections from subcutaneously xenotransplanted tumors. Scale bar 10 μm. **F**, **G** Immunofluorescent staining against Ki67 (**F**) and TUNEL (**G**) of the tumor sections, scale bar 50 μm. **H** IHC staining against p-PKA or RNF2 of the subcutaneously xenotransplanted tumor. Scale bar 50 μm. **I** A diagram depicting the working model of the inhibitory effect of GPR17 on glioma tumorigenesis. For all panels, *p < 0.05, ***p < 0.001, Student’s *t-*test.

The inhibitory effects of GPR17 on glioma cell viability make it a potential therapeutic target for glioma. To test the possibility, we used MDL29951, a GPR17 agonist [54], to investigate whether activation of GPR17 showed inhibitory effects on glioma development. We first administrated MDL29951 into U87MG and U251 cells and found that it significantly suppressed viable cells numbers (Fig. 7A). Given the fact that we had no evidence that MDL29951 could cross blood–brain barrier, we generated a subcutaneous xenotransplanted tumor model in BALB/c nude mice, and treated the mice with MDL29951 for 16 days starting from day 7 after transplantation, to test whether MDL29951 inhibited glioma tumorigenesis. As shown, MDL29951 treatment significantly inhibited tumor growth (Fig. 7B–D). Furthermore, H&E and immunofluorescent imaging against Ki67 or TUNEL indicated that MDL29951 inhibited cell proliferation and induced cell death (Fig. 7E–G). Besides, the protein levels of p-PKA and RNF2 were both significantly decreased in U87 xenografts after MDL29951 treatment, which further revealed the role of GPR17 activation in the control of PKA signaling activity and *RNF2* expression in glioma (Fig. 7H). These data suggested that pharmacologically activating GPR17 was beneficial for suppressing glioma formation.

## Discussion

Glioma is one of the most lethal tumors in the central nervous system [3–5]. Developing practical pharmacological interventions is crucial for glioma therapy. In this study, we found that GPR17 was a potential therapeutic target against glioma, as either overexpression or pharmacological activation of GPR17 effectively inhibited glioma tumorigenesis.

At the beginning of our study, to comprehensively understand the transcriptomic alteration during glioma development, we performed data mining with the LGG and GBM patient samples in TCGA and CGGA database. According to our analysis, GPR17 was one of GPCRs with the most significant differential expression patterns. Consistent with our analysis, Mutharasu et al. [18] reported that GPR17 interacted with about 30 different crucial pathways in glioblastoma multiforme signaling networks, and suggested that GPR17 mediated signaling networks could be used as a therapeutic target for GBM. Notably, so far, several GPR17 agonists or antagonists have been established [54], which will greatly facilitate the application of targeting GPR17 for glioma therapy. Therefore, we paid our efforts to study the role of GPR17 during glioma development, and sought to evaluate its therapeutic potential for glioma.

To date, GPR17 has been implicated to play roles in glioma development. Dougherty et al. [19] reported that GPR17 agonists treatment decreased the number of neurospheres of primary murine glioblastoma cells. However, the mechanism about the molecular functions of GPR17 in giloma cells remained unknown. In our present study, using multiple models and approaches, we demonstrated a GPR17–RNF2–KLF9 regulatory axis that controlled cell survival by modulating ROS level in glioma cells.

The augmented ROS level was the key signature for the effect of GPR17 on glioma cell death, as the elimination of excessive ROS by NAC abolished the effects of GPR17. ROS is known to played pivotal roles in the control of glioma formation [55]. In this study, we found that GPR17 increased ROS level in glioma cells through a regulation on SOD1 gene expression. Recently, Wang et al. [56] reported that antagonism of GPR17 by its antagonist pranlukast decreased the intracellular ROS level in a chondrogenic cell line, by increasing the activity of the SODs against TNFα, which were consistent with our finding and further corroborated the effect of GPR17 on intracellular ROS level.

In the regulatory axis of GPR17 on intracellular ROS level, RNF2 and KLF9 were two key mediators. Previously, a polycomb repressive complex component, Bmi1, has been found to regulate the intracellular ROS level [57, 58]. In our study, we found that RNF2, another key component of the PRC1 complex, mediated the effects of GPR17 on intracellular ROS level. So far, the molecular basis underpinning the connection between PRC1 complex and intracellular ROS level remained elusive. Here, we identified KLF9 as the key transcription factor that connecting PRC1 complex and histone H2A subunit monoubiquitination to ROS level. A combination of RNA-Seq and ChIP-Seq analyses indicated that RNF2 inhibited *KLF9* gene expression through its recruitment to *KLF9* promoter region. As reported, KLF9 elevated intracellular ROS level by reducing the expressions of the reductases, resulting in its impacts on tumor cell proliferation, apoptosis, and metastasis [46, 59–62]. In glioma cells, KLF9 was identified to suppress the expression of *SOD1*, accomplishing the regulatory effects of GPR17 on ROS level.

Taken together, we proposed a working model to summarize our findings as shown in Fig. 7I. The activation of GPR17 reduced intracellular cAMP level, and then inhibited PKA signaling activity. The inactivation of PKA signaling suppressed the phosphorylation and activation of p65, resulting in a reduction of *RNF2* expression and the subsequent PRC1-mediated histone H2A K119 monoubiquitination on *KLF9* promoter. As a result, the expression of *KLF9* was elevated, leading to the augmented ROS level, which eventually inhibited cell proliferation and induced apoptosis.

## Supplementary information

supplementary table

supplementary figures

## Data Availability

All original data presented in this study are available upon reasonable request. The high-throughput sequencing data have been uploaded to GEO (GSE174123).
