# Supplementary material for: G-protein-coupled receptor GPR17 inhibits glioma development by increasing polycomb repressive complex 1-mediated ROS production
Source: Cell Death Dis. 2021 Jun 12;12(6):610. doi: 10.1038/s41419-021-03897-0 (PMC8197764; doi:10.1038/s41419-021-03897-0)
Supplement: Supplementary file 1 — supplementary table [file 41419_2021_3897_MOESM1_ESM.docx]

**Table S1 Primers and shRNA sequences used in this study**

**Primers for qRT-PCR**

| Primer name | Primers sequences 5′-3′ |
| --- | --- |
| GPR17-Forward | TACCACTTCTCTGGGAACCACT |
| GPR17-Reverse | CTGGCGTACATGTTGAGGTAGA |
| RNF2-Forward | ATCCAAGTATCTGGCTGTGAGG |
| RNF2-Reverse | GCTTCTCACTGGCTGTATCAAG |
| GAPDH-Forward | GACATCAAGAAGGTGGTGAAGC |
| GAPDH-Reverse | GCTGTTGAAGTCAGAGGAGACC |
| KLF9-Forward | ACTGCACACTGGTCACCATC |
| KLF9-Reverse | TGGACTTTCCAGACTGTCGC |
| SOD1-Forward | CATTGCATCATTGGCCGCA |
| SOD1-Reverse | CCACAAGCCAAACGACTTCC |
| SOD2-Forward | AGAGGTCTGCATTATGCTTGC |
| SOD2-Reverse | CCTTTCCCATGGAAACTCAGTG |
| GSR-Forward | GTGGAACACAGCTGTCCACT |
| GSR-Reverse | TCACATAGGCATCCCGCTTT |
| GPX1-Forward | CCAGTTTGGGCATCAGGAGAA |
| GPX1-Reverse | AGCATGAAGTTGGGCTCGAA |
| GPX3-Forward | AGAAGTCGAAGATGGACTGCC |
| GPX3-Reverse | GGGATGTACTCCTCCCCATCA |
| GSTP1-Forward | GCTCTATGGGAAGGACCAGC |
| GSTP1-Reverse | CCCGCCTCATAGTTGGTGTA |
| PRDX1-Forward | GCTAAAATTGGGCACCCTGC |
| PRDX1-Reverse | TCCTTTGTAGTCAGACAGGCT |
| PRDX3-Forward | CGAGACTACGGTGTGCTGTT |
| PRDX3-Reverse | CCCACTGGGAGATCGTTGAC |
| PARK7-Forward | AGATGTCATGAGGCGAGCTG |
| PARK7-Reverse | TGACCACATCACGGCTACAC |
| NQO1-Forward | CTGAAGGACCCTGCGAACTT |
| NQO1-Reverse | TATCACAAGGTCTGCGGCTT |
| GAS6-Forward | CAGGACCTCATGGGCAACTT |
| GAS6-Reverse | TTCTCCTGGCTGCATTCGTT |
| KCNH1-Forward | TTGTTCGGCGGTCCAATGAT |
| KCNH1-Reverse | CCCTGTGATAGCCAGACAGC |
| LBH-Forward | ATATTTCCCCATTCACTGCCCC |
| LBH-Reverse | TAGGAAAGGCCATCCTTGCG |
| TMOD1-Forward | CCTTGGAGCCCTAACAGAGG |
| TMOD1-Reverse | GCAGGCAGCAGTGCATTATC |
| C5orf46-Forward | AAATTCCTAAGCCTCCTGGGC |
| C5orf46-Reverse | ATTCCATAAATCCTGTGCTCCT |
| PTK7-Forward | CTCCCTGGTGATCCATGACG |
| PTK7-Reverse | TTGATGTTGCAGCTGTTGCC |
| TMEM268-Forward | ATCCCAGGGCTGGAATCTCT |
| TMEM268-Reverse | CGAGATCTCCTTGCACCACA |

**Primers for ChIP assay**

| Primer name | Primers sequences 5′-3′ |
| --- | --- |
| KLF9 CHIP-Forward | TTCTCTTCCCTCACTTCACAAATG |
| KLF9 CHIP-Reverse | GCTAGAAATCAGGAGACTTGTGGT |

**Primers for shRNA**

| Primer name | Primers sequences 5′-3′ |
| --- | --- |
| ShGPR17#1-F | CCGGCCTCACCTGCATCAGCGCCGACTCGAGTCGGCGCTGATGCAGGTGAGGTTTTTG |
| ShGPR17#1-R | AATTCAAAAACCTCACCTGCATCAGCGCCGACTCGAG TTGTCTCGGCGCTGATGCAGGTGAGG |
| ShGPR17#2-F | CCGGGGCCCTGGCAAACCGCATCACCTCGAGGTGATGCGGTTTGCCAGGGCC |
| ShGPR17#2-R | AATTCAAAAAGGCCCTGGCAAACCGCATCACCTCGAGTGATGCGGTTTGCCAGGGCC |
| ShRNF1#1-F | CCGGAGACGAGGTATGTGAAGACAACTCGAGTTGTCTTCACATACCTCGTCT TTTTTG |
| ShRNF1#1-R | AATTCAAAAAAGACGAGGTATGTGAAGACAACTCGAG TTGTCTTCACATACCTCGTCT |
| ShRNF1#2-F | CCGGCTGGAGCTGGTGAATGAGAAACTCGAGTTTCTCATTCACCAGCTCCAG TTTTTG |
| ShRNF1#2-R | AATTCAAAAACTGGAGCTGGTGAATGAGAAACTCGAG TTTCTCATTCACCAGCTCCAG |
| ShRNF2#1-F | CCGGGCCAGGATCAACAAGCACAATCTCGAGATTGTGCTTGTTGATCCTGGC TTTTTG |
| ShRNF2#1-R | AATTCAAAAAGCCAGGATCAACAAGCACAATCTCGAG ATTGTGCTTGTTGATCCTGGC |
| ShRNF2#2-F | CCGGGCACCTACAAAGGAGCACAAACTCGAGTTTGTGCTCCTTTGTAGGTGC TTTTTG |
| ShRNF2#2-R | AATTCAAAAAGCACCTACAAAGGAGCACAAACTCGAG TTTGTGCTCCTTTGTAGGTGC |
| ShKLF9#1-F | CCGGGCCCAGTGTCTGGTTTCCATTCTCGAGAATGGAAACCAGACACTGGGCTTTTTG |
| ShKLF9#1-R | AATTCAAAAAGCCCAGTGTCTGGTTTCCATTCTCGAGAATGGAAACCAGACACTGGGC |
| ShKLF9#2-F | CCGGGGAAGGATTACTGCACACTGGCTCGAGCCAGTGTGCAGTAATCCTTCCTTTTTG |
| ShKLF9#2-R | AATTCAAAAAGGAAGGATTACTGCACACTGGCTCGAGCCAGTGTGCAGTAATCCTTCC |
