# Supplementary material for: G-protein-coupled receptor GPR17 inhibits glioma development by increasing polycomb repressive complex 1-mediated ROS production
Source: Cell Death Dis. 2021 Jun 12;12(6):610. doi: 10.1038/s41419-021-03897-0 (PMC8197764; doi:10.1038/s41419-021-03897-0)
Supplement: Supplementary file 2 — supplementary figures [file 41419_2021_3897_MOESM2_ESM.pdf]

Supplementary Figures

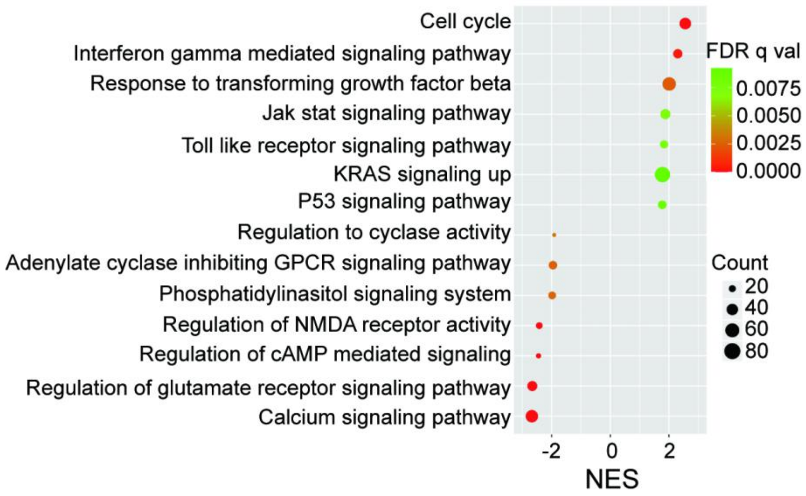

Figure S1. GSEA analysis of LGGs and GBMs for top differentially regulated gene sets

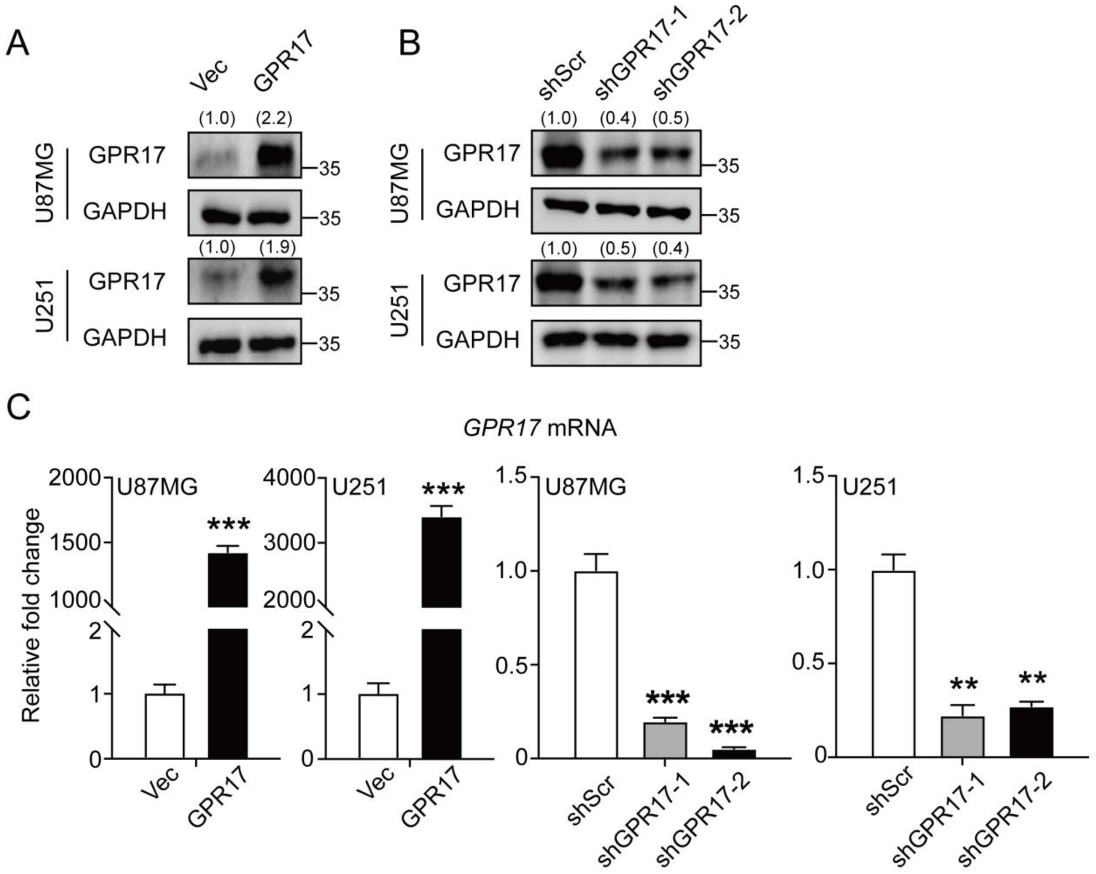

Figure S2. Confirmation of GPR17 overexpression or knockdown efficiency in the stable cell lines generated from U87MG or U251 cells

Control, U87/U251-GPR17 or U87/U251-shGPR17 cells were prepared as in Figure 2. (A and B) Cells were harvested for Western blot to detect the protein level of GPR17. Densitometric quantification of GPR17/GAPDH ratio from at least 3 independent assays was indicated on top of each band, respectively. (C) Cells were harvested for real-time PCR detecting the mRNA level of GPR17. Data represent the means ± SEM from three independent experiments. **p < 0.01, ***p < 0.001, Student's t test.

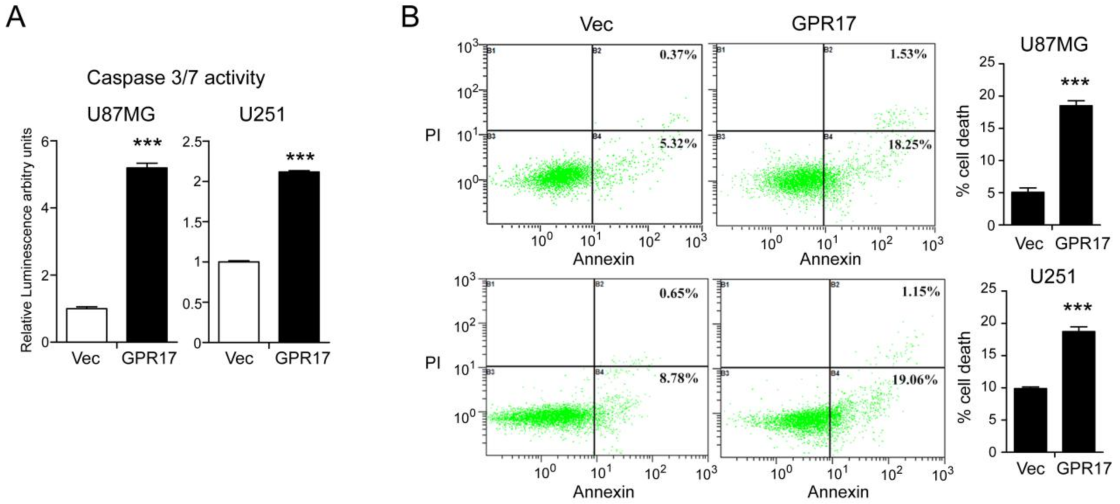

Figure S3. GPR17 induced apoptotic cell death

(A, B) Control and U87/U251-GPR17 cells were prepared as in Figure 2. Cells were harvested for Caspase 3/7 activities (A), and for flow cytometry analysis on cell death (B). Data represent the means ± SEM from 3 independent experiments. ***$p < 0.001$, Student's t test.

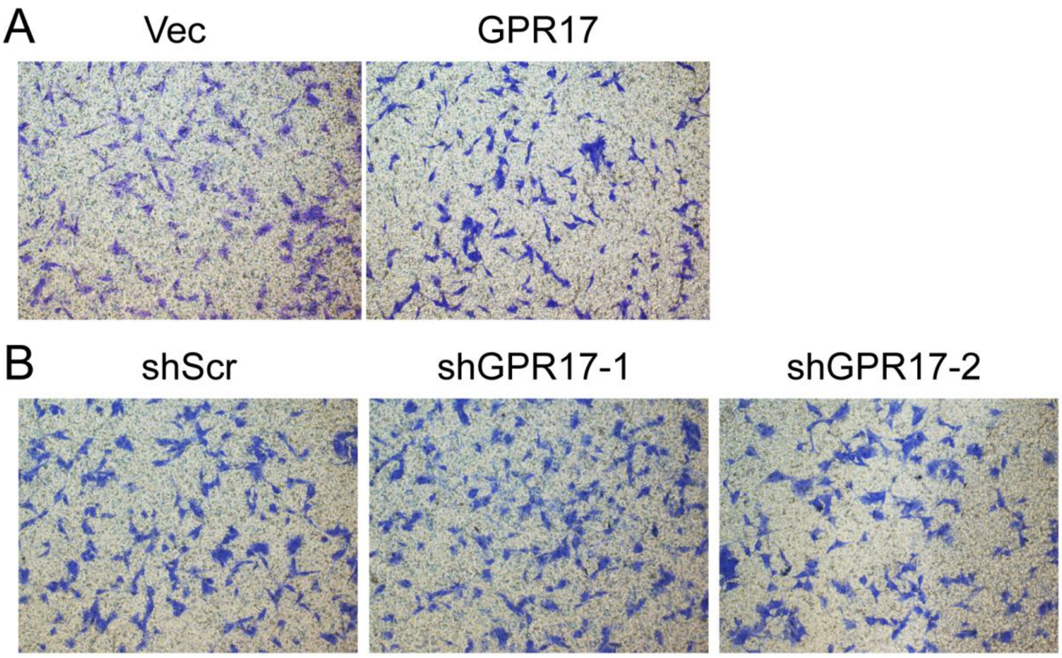

Figure S4. GPR17 did not affect glioma cell invasion

(A, B) Control, U87-GPR17 or U87-shGPR17 cells were prepared as in Figure 2. Cells were seeded in medium without FBS in the upper compartment of transwell chambers and the lower chamber was filled with medium with 10% FBS for 24 h. The bottom sides of the filters were stained with crystal violet to count the cells that migrated across the filter.

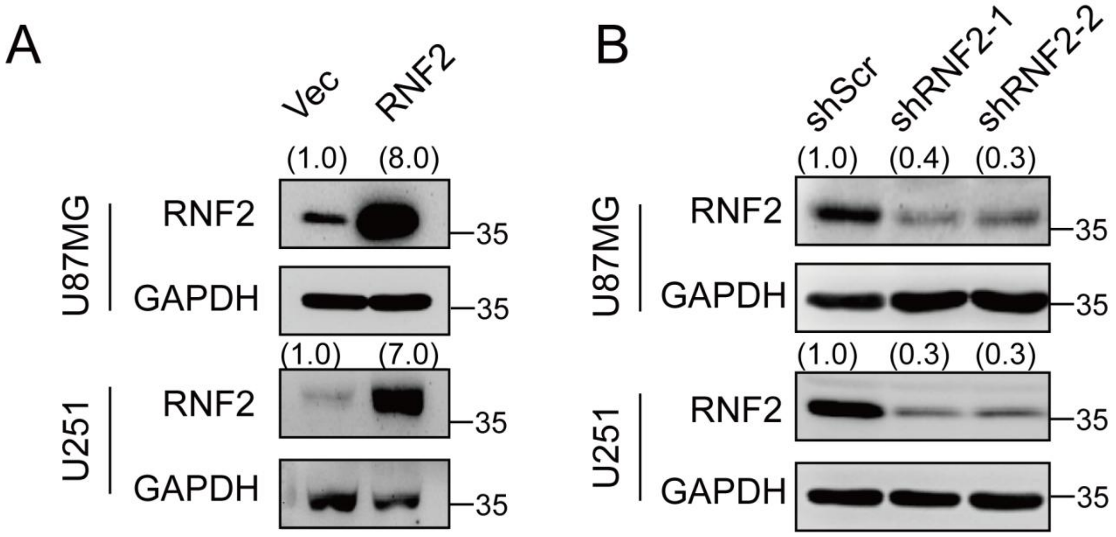

Figure S5. Confirmation of RNF2 overexpression or knockdown efficiency in the stable cell lines generated from U87MG or U251 cells

Control, U87/U251-RNF2 or U87/U251-shRNF2 cells were prepared as in Figure 3. Cells were harvested for Western blot to detect the protein level of RNF2. Densitometric quantification of RNF2/GAPDH ratio from at least 3 independent assays was indicated on top of each band, respectively.

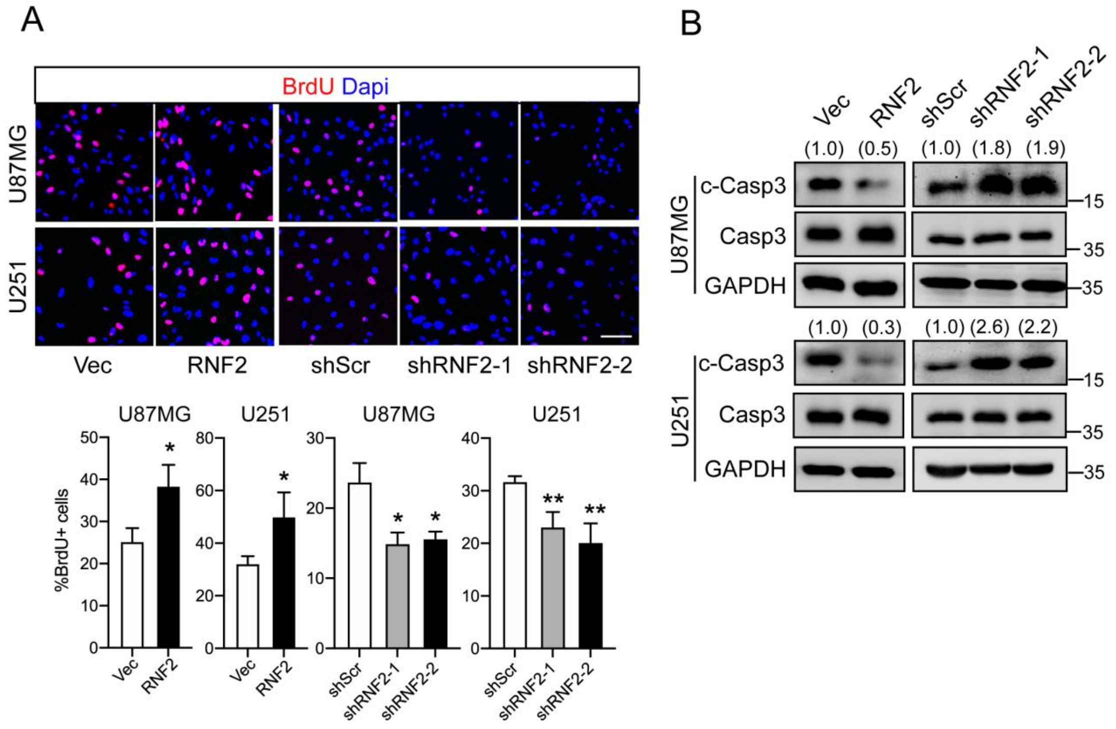

Figure S6. RNF2 induced cell proliferation, but inhibited apoptosis in glioma cells

Control, U87/U251-RNF2 or U87/U251-shRNF2 cells were prepared as in Figure 3. Cells were harvested for immunofluorescent staining against BrdU (A), and for western blot against cleaved-caspase3 (B). Densitometric quantification of cleaved-caspase3/caspase3 ratio from at least 3 independent assays was indicated on top of each band, respectively.

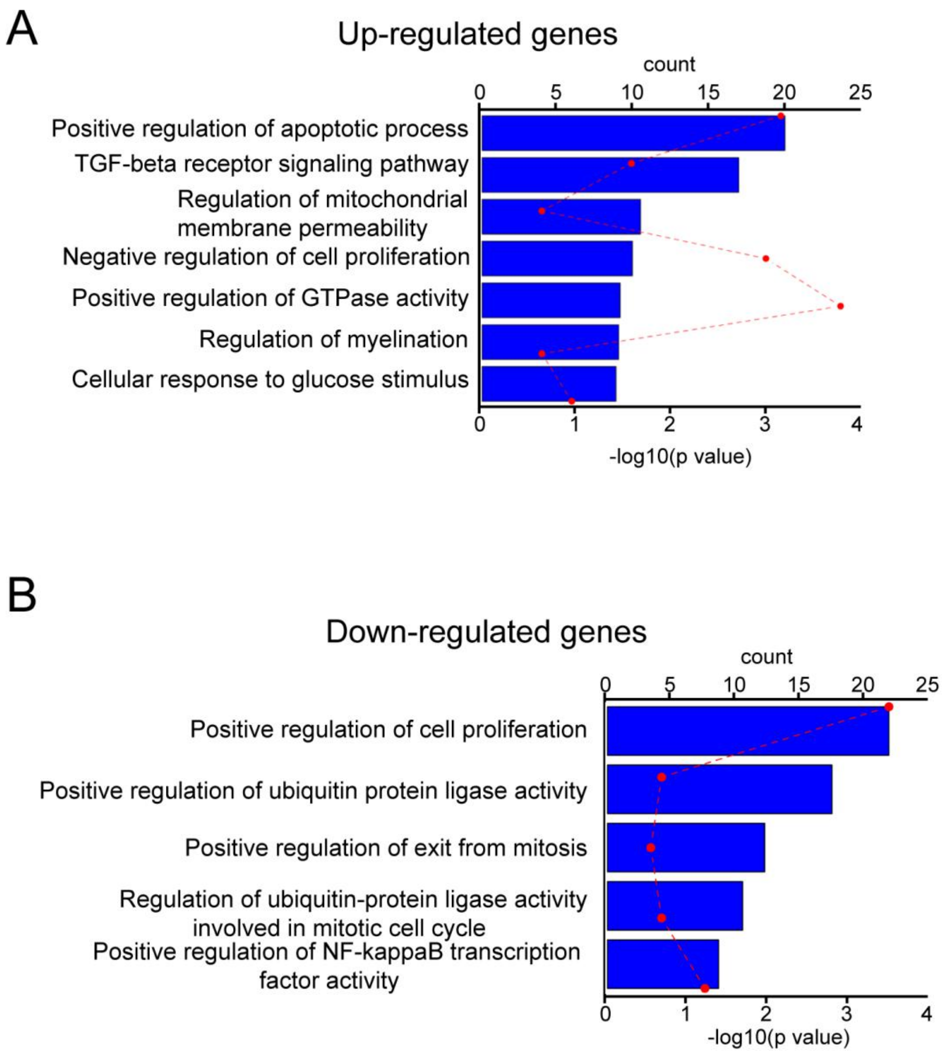

Figure S7. The gene ontology (GO) analysis of the U87-Vec/U87-GPR17 cells RNA-Seq

(A, B) The gene ontology (GO) analysis of the significantly up-regulated (A) and down-regulated genes (B) in U87-GPR17 cells, compared to U87-Vec cells.

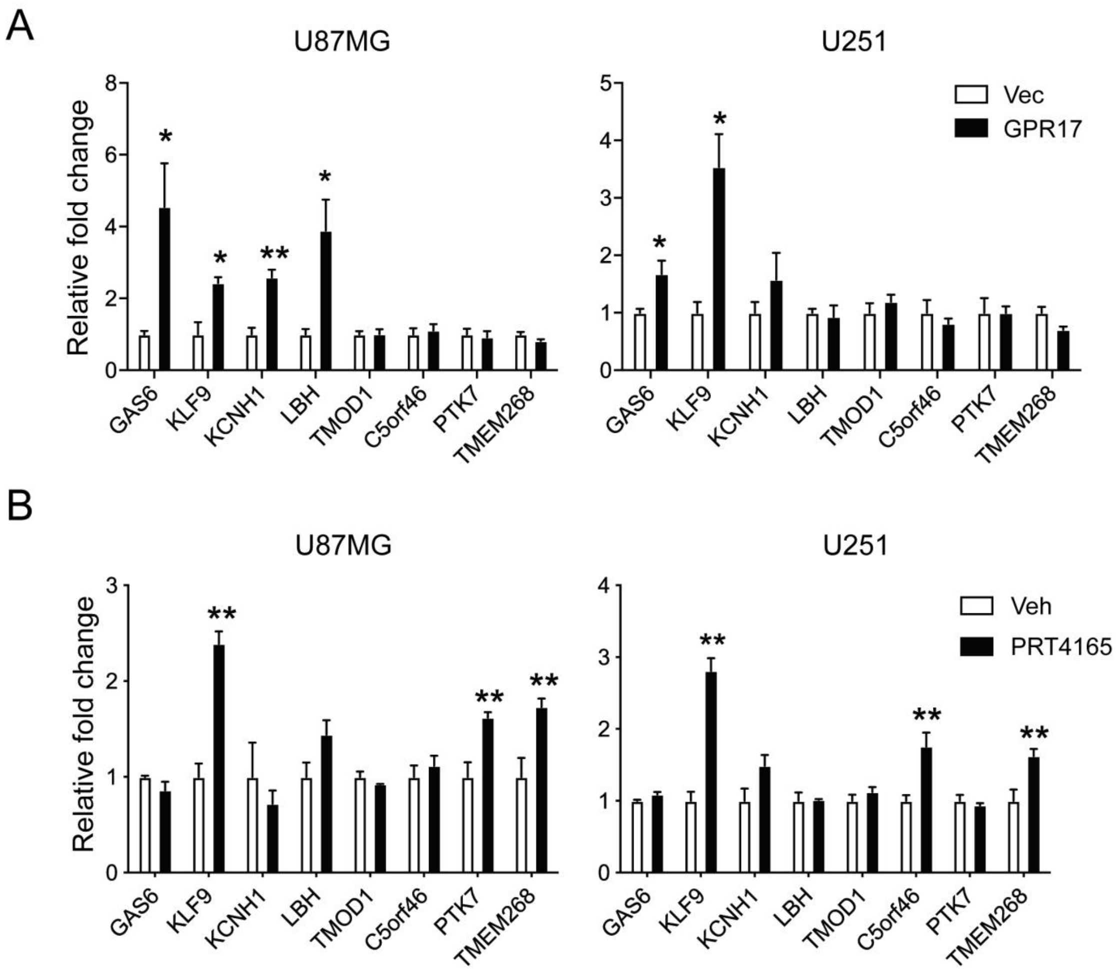

Figure S8. KLF9 was the potential downstream candidate target for GPR17 and RNF2

(A) Control, U87/U251-GPR17 cells were prepared as in Figure2. Cells were harvested for real-time PCR analysis to assess the mRNA levels of the indicated genes.(B) U87MG or U251 cells were treated with 10 μM PRT4165 for 48 h. Cells were then harvested for real-time PCR analysis to assess the mRNA levels of the indicated genes.
